# A six year retrospective review of occipital nerve stimulation practice - controversies and challenges of an emerging technique for treating refractory headache syndromes

**DOI:** 10.1186/1129-2377-14-67

**Published:** 2013-08-06

**Authors:** Stefano Palmisani, Adnan Al-Kaisy, Roberto Arcioni, Tom Smith, Andrea Negro, Giorgio Lambru, Vijay Bandikatla, Eleanor Carson, Paolo Martelletti

**Affiliations:** 1 Pain Management & Neuromodulation Centre, Guy’s & St Thomas NHS Trust, London, UK; 2 Department of Medical and Surgical Science and Translational Medicine, Sapienza University of Rome and Pain Therapy Unit Sant’Andrea Hospital, Rome, Italy; 3 Department of Clinical and Molecular Medicine, Sant’Andrea Hospital, Sapienza University of Rome and Regional Referral Headache Centre, Rome, Italy

**Keywords:** Headache, Chronic migraine, Occipital neuralgia, Neuromodulation, Occipital nerve stimulation

## Abstract

**Background:**

A retrospective review of patients treated with Occipital Nerve Stimulation (ONS) at two large tertiary referral centres has been audited in order to optimise future treatment pathways.

**Methods:**

Patient’s medical records were retrospectively reviewed, and each patient was contacted by a trained headache expert to confirm clinical diagnosis and system efficacy. Results were compared to reported outcomes in current literature on ONS for primary headaches.

**Results:**

Twenty-five patients underwent a trial of ONS between January 2007 and December 2012, and 23 patients went on to have permanent implantation of ONS. All 23 patients reached one-year follow/up, and 14 of them (61%) exceeded two years of follow-up. Seventeen of the 23 had refractory chronic migraine (rCM), and 3 refractory occipital neuralgia (ON). 11 of the 19 rCM patients had been referred with an incorrect headache diagnosis. Nine of the rCM patients (53%) reported 50% or more reduction in headache pain intensity and or frequency at long term follow-up (11–77 months). All 3 ON patients reported more than 50% reduction in pain intensity and/or frequency at 28–31 months. Ten (43%) subjects underwent surgical revision after an average of 11 ± 7 months from permanent implantation - in 90% of cases due to lead problems. Seven patients attended a specifically designed, multi-disciplinary, two-week pre-implant programme and showed improved scores across all measured psychological and functional parameters independent of response to subsequent ONS.

**Conclusions:**

Our retrospective review: 1) confirms the long-term ONS success rate in refractory chronic headaches, consistent with previously published studies; 2) suggests that some headaches types may respond better to ONS than others (ON vs CM); 3) calls into question the role of trial stimulation in ONS; 4) confirms the high rate of complications related to the equipment not originally designed for ONS; 5) emphasises the need for specialist multidisciplinary care in these patients.

## Background

Chronic Daily Headache (CDH) is an umbrella term for headache disorders with a high rate of reoccurrence (15 or more days per month for 3 consecutive months). CDH represents a major worldwide health problem as affects 3–5% of adults [1-3] who experience substantial disability.

Chronic migraine (CM), the most prevalent form of CDH, is defined as headache occurring more than 15 days/month for at least 3 consecutive months, with headache having the clinical features of migraine without aura for at least 8 days per month [4]. Recently published results from the American Migraine Prevalence and Prevention Study (AMPP) found the prevalence of CM in the United States is approximately 1% [5]. The World Health Organization recognizes migraine as a major public health problem, ranking it at 7th place among all worldwide diseases leading to disability [6]. Compared to episodic migraine, CM is associated with higher disability, inferior quality of life and greater health resource utilization [7].

Despite substantial advances in migraine therapy [8], some individuals with chronic migraine are either resistant or intolerant to guideline-based treatments [9]. This subset of patients requires the development of further treatments and in recent years peripheral neuromodulation, in the form of occipital nerve stimulation (ONS), has emerged as an option for this subset of patients [8,10]. Several published small retrospective studies reported promising safety and efficacy data for ONS in primary headaches.

Open label studies in trigeminal autonomic cephalalgias have shown significant, long-term benefit in 67% of refractory chronic cluster headache patients [10] and in 89% of refractory SUNCT and SUNA (short-lasting neuralgiform headache attacks with conjunctival injection and tearing/autonomic symptoms) patients [11]. Encouraging results in refractory chronic migraine patients led to three, commercially funded, multi-centre randomized trials [12,13]. The benefits shown in these trials were less dramatic than hoped for, however the studies have been criticised for methodological weaknesses, unmitigated placebo effect, and a high rate of surgical complications, which may have obscured the full beneficial effect of ONS. Limited data on relevant endpoints was available at the time of studies’ design and poor endpoint choice may have masked the true efficacy of ONS [13].

Thus, the literature leaves many questions unanswered about the role for ONS in chronic daily headache. Our institutions are large, tertiary neuromodulation centers with a special interest in headaches. We agreed to pool resources and retrospectively audit our own data on ONS for CDH to help guide us on future clinical indications for ONS, identify areas for improved clinical practice, technical practice and data collection.

This paper reports the results of our audit and relates these to the literature. We discuss the importance of specialists within a multidisciplinary treatment team, question the use of temporary trials to select ONS-responders, and look at surgical strategies to limit hardware-related complications.

## Methods

Two large tertiary neuromodulation centers (Guy’s & St Thomas NHS Trust, London, United Kingdom and Sapienza University at Sant’Andrea Hospital, Rome, Italy) retrospectively audited outcomes of patients receiving ONS from the previous 6 years. The audit results were analyzed with reference to available literature on ONS for CDH. Ethics committee approval was not required for this audit.

### Audit process

All patients receiving a trial of ONS in the last 6 years at both institutions were included in the audit. Patient demographics, headache phenotype and technical details of the surgical procedure(s) were collected from patient medical records. Telephone reviews (up to three per patient) were performed by one headache specialist for each site (GL and PM) to confirm data accuracy, system efficacy and, when needed, to re-code patients’ diagnosis according to the ICHD-II classification [14].

### ONS indication

At both sites, the indication for ONS was refractory chronic headaches. Patients had failed to significantly improve after adequate trials of four classes of preventive medicines and three classes of acute drugs with established efficacy [15].

ONS candidates were advised not to proceed with surgery when psychological evaluation identified conditions which could be aggravated by the treatment or cause confusion in interpreting clinical results (including, but not limited to, intractable epilepsy, active major depression, psychosis, somatoform disorder, severe personality disorder).

### Surgical procedure

**Figure 1:**
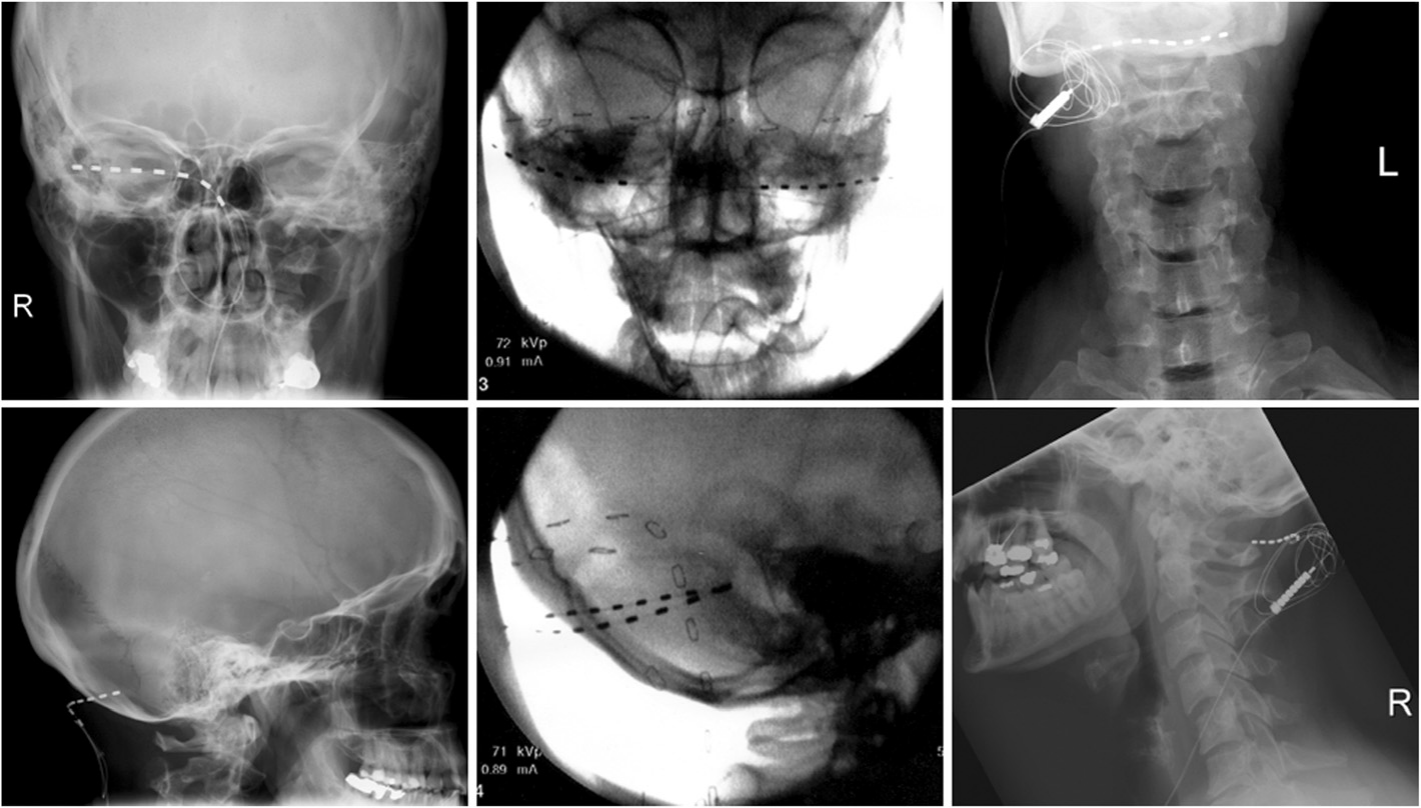
**Example of 3 different approaches for ONS.** From left to right, 1) single lead monolateral ONS; 2) dual lead, bilateral ONS; 3) single lead bilateral ONS.

The ONS surgical procedure was performed by four different operators, with equipment and surgical technique (particularly lead insertion and anchoring) varying between operators and over time (Figure 1). All patients underwent a trial of therapy. One or two percutaneous lead(s) were inserted under sedation in the subcutaneous tissue above the peripheral branches of the occipital nerves at approximately C1 level, and left in place for 7 – 10 days to evaluate the efficacy and tolerability of the treatment before being removed. If the trial was “successful”, i.e. the patient reported at least 50% decrease in headache intensity and/or frequency associated with a decrease headache medication use, a permanent implant was then performed under general anaesthesia. Leads were implanted as in the trial, but this time they were anchored to fascia, tunnelled, and connected to an IPG sited in a subcutaneous abdominal pocket. The practice of leaving stress relief loops in each of the subcutaneous incisions was implemented in some of the subjects implanted after the technique was widely published as part of large multi-centre study [16].

### Outcome

The patients were treated by different physicians in different centres across a 6-year timeframe and a variety of outcomes were measured for both trial and full implant efficacy. To homogenously evaluate ONS outcomes and be consistent in neuromodulation trial evaluation, we decided a patient implanted with a permanent ONS system would be considered a “success” if a sustained decrease of at least 50% in headache intensity and/or frequency was reported by the patient during the telephone review. Those patients with whom we did not make telephone contact were excluded from the outcome analysis regardless of the information reported in their medical notes.

### Cognitive Behavioural Therapy (CBT) based implant preparation

In one of the two centres involved in the study (GSTT), some patients were required to attend a pre-implant programme (PIP) before proceeding to the trial stage. The PIP involves groups of up to 11 patients engaging in 7–9 days activity spread over two weeks. Physicians, psychologists, physiotherapists, nurses and occupational therapists provide a variety of broadly CBT based interventions, which explicitly seek to reduce emotional distress [17] and improve social and physical functioning [18]. This is done by addressing an individual’s interpretation, evaluations and beliefs about their health condition [19].

Several outcome measures are routinely collected during the course of the PIP, many of those reflecting the IMMPACT recommendations [20] and measuring pain-related disability as a primary outcome variable. Among those: 1) The Pain Disability Index (PDI), which measures the extent to which chronic pain interferes with daily activities [21]; 2) the Beck Depression Index (BDI), which measures the severity of self-reported depressive symptoms [22]; 3) the Pain Self Efficacy Questionnaire (PSEQ), which evaluates how confident patients feel about carry out a variety of tasks despite their pain [23]; 4) the Pain Catastrophizing Scale (PCS), which measures the extent of catastrophising thoughts and feelings associated with pain [24]; 5) the Tampa Scale of Kinesiophobia (TSK), which is a measure of pain-related fear of movement or re-injury [25].

Patients at GSTT who did not do a PIP attended a “Technology Day” instead. This examines patient expectations of treatment with a psychologist, includes information and question and answer sessions given by a physiotherapist and nurse on the stimulator itself and briefly educates on chronic pain and ways of managing this more effectively. Formal psychological data is not gathered, and CBT-based interventions are not provided.

### Statistics

Descriptive statistics has been used to interpret data as appropriate, and data were presented mean ± standard deviation if not stated otherwise. Wilcoxon signed-rank non-parametric test has been used to compare psychological variables in the small subgroup of patients who attended the PIP. Significance level was set at α = 0.05.

## Results

Twenty-five patients underwent a trial of ONS between January 2007 and December 2012 (Male/Female: 7/18; Average age: 49 ± 14 years) (Table 1). Only three patients did not report enough relief during the period of percutaneous stimulation to consider the trial a successful (success rate = 88%), but one patient still requested and received a permanent system. Therefore, 23 patients who received a permanent ONS system were included in the following analysis (Table 2).

**Table 1:** Diagnoses, laterality and site of the pain of the sample of headache patients trialled with Occipital nerve stimulation

		**Diagnosis**					**Pain distribution**	
	**Sex**	**Definitive**	**Preliminary**	**Trigger**	**Bilateral**	**Length**	**Area of origin**	**Radiation**
1	F	CM	ON	No	Y	2 ys	Occipital	Vertex
2*	F	CM	CM	No	Y	16 ys	Neck/Occipital	Occipital
3	F	CM	ON	Yes (S)	Y	6 ys	Occipital	Forehead
4	F	CM	CDH	Yes (S)	Y	N/A	Ear	Ear/Face
5	F	CM	Migraine	No	N	15 ys	Eye	Eye
6*	M	CM	ON	No	N	17 ys	Eye	Forehead
7	F	CM	ON	Yes (T)	Y	1 ys	Occipital	Vertex
8	F	IIH	ON	No	Y	2 ys	Occipital	Holocranic
9*	M	CM	ON	No	Y	8 ys	Neck	Occipital
10	M	ON	ON	No	Y	10 ys	Occipital	Holocranic
11	F	ON	ON	No	Y	4 ys	Neck/Occipital	Vertex
12	F	CH	CH	No	N	5 ys	Occipital	Eye
13	M	CM	ON	No	N	N/A	N/A	N/A
14	F	ON	ON	No	Y	3 ys	Occipital	Shoulders
15	F	CM	ON	No	Y	15 ys	Neck	Temple
16	M	CM	Migraine	No	N	3 ys	Occipital	Hemicranium
17	M	CM	ON	No	Y	15 ys	Neck/Occipital	Forehead
18	F	CM	ON	No	Y	15 ys	Neck/Occipital	Eye
19	F	CM	ON	Yes (T)	Y	19 ys	Occipital	Forehead
20	M	Cerv.H.	ON	Yes (T)	Y	10 ys	Occipital	Forehead
21	F	CM	ON	Yes (T)	N	3 ys	Neck	Vertex/Eye
22	F	CM	CM	N/A	N	N/A	Temple	Temple
23	F	CM	CM	N/A	N	N/A	Temple	Forehead
24	F	CM	CM	N/A	N	N/A	Eye	Hemicranium
25	F	CM	CM	N/A	Y	N/A	Forehead	Holocranic

*CM* chronic migraine, *IIH* idiopathic intracranial hypertension, *ON* occipital neuralgia, *CH* Cluster Headache, *CDH* chronic daily headache, *Cerv.H* Cervicogenic Headache, *N/A* data not available, *T* Post-traumatic, onset of the headache within a week following a head/neck injury, *S* Post-Surgical, onset of the headache within a week from a scheduled or un-scheduled surgery; * Pts considered to have failed the ONS trial.

**Table 2:** Paraesthesia coverage, types of implants, outcome, complications and removal rate of patients implanted with occipital nerve stimulation

	**Diagnosis**	**Side shift**	**Origin of pain**	**Implant success**	**Lead(s)**	**Paraesthesia coverage**	**Last Fw/up**	**Revision surgery**	**Time to revision**	**Removal**	**Time to removal**
*1*	CM	Y	Occipital	No	1 Quadripolar	Good	77	--	--	--	--
*2*	CM	Y	Neck/ Occipital	No	1 Quadripolar	Excellent	--	--	--	Painful paraesthesia - inefficacy	10
*3*	CM	Y	Occipital	Yes (100%)	2 Quadripolar	Good	71	Battery site hyperalgesia	2	--	--
*4*	CM	Y	Ear	Yes (90%)	2 Octopolar	Moderate	--	--	--	Granuloma and skin erosion	29
*5*	CM	N	Eye	Yes (100%)	2 Octopolar	Moderate	42	--	--	--	--
*6*	CM	Y	Occipital	Yes (90%)	2 Octopolar	Excellent	18	Infection lead (×2)	11	--	--
*7*	IIH	Y	Occipital	Yes (100%)	2 Octopolar	Excellent	21	--	--	--	--
*8*	ON	Y	Occipital	Yes (100%)	1 Quadripolar	Excellent	28	--	--	--	--
*9*	ON	Y	Neck/ Occipital	Yes (70%)	N/A	Excellent	31	Tilted IPG	N/A	--	--
*10*	CH	N	Occipital	Yes (50%)	N/A	N/A	28	Lead replacement (High Imp.)	24	--	--
*11*	CM	N	N/A	No (<50%)	2 Octopolar	Poor	--	--	--	Inefficacy	54
*12*	ON	Y	Occipital	Yes (50%)	N/A	Good	28	--	--	--	--
*13*	CM	Y	Neck	Yes (100%)	N/A	Excellent	48	Skin erosion (×3)	N/A	--	--
*14*	CM	N	Occipital	No	N/A	N/A	--	--	--	Inefficacy and implant site infection	2
*15*	CM	Y	Neck/ Occipital	No	2 Octopolar	Excellent	--	1st: painful paraesthesia; 2nd: SO lead added	12	Inefficacy	35
*16*	CM	Y	Neck/ Occipital	No	1 Octopolar	Excellent	79	--	--	--	--
*17*	CM	Y	Occipital	No	1 Octopolar	Excellent	--	Lead migration	8	Inefficacy	20
*18*	Cerv.H.	Y	Occipital	No	2 Octopolar	Good	--	Several granulomas, lead breakage	N/A	Inefficacy and implant site infection	61
*19*	CM	N	Neck	Yes (50%)	2 Octopolar	Moderate	31	--	--	--	--
*20*	CM	N	Temple	Yes (70%)	2 Quadripolar	Poor	13	--	--	--	--
*21*	CM	N	Temple	Yes (50%)	2 Quadripolar	Poor	11	Lead and IPG replaced (High Imp.) SO lead added	7	--	--
*22*	CM	N	Eye	Yes (50%)	2 Quadripolar	Poor	12	--	--	--	--
*23*	CM	Y	Forehead	No	2 Quadripolar	Poor	--	Lead migration	--	Pt request despite effective	12

*FI* full implant, *IPG* implanted pulse generator, *N/A* data not available, *SO* supraorbital, *Paresthesia Coverage* % of original painful area covered by paresthesia. Last follow/up (Fw/up), Time to Revision and Time to removal all expressed in months.

All patients reached one-year follow-up, and 14 of them (61%) exceeded two years of follow-up (Average 36 ± 23 months, median 28 months). Ten (43%) subjects underwent at least one surgical revision after an average of 11 ± 7 months from permanent implantation, and 90% of the revision surgeries were needed because of problems with leads. Battery replacements were not considered as surgical revisions, unless battery depletion was caused by high lead impedances. Nine patients required at least one surgical revision to replace the stimulating lead because of displacement (3), high impedances (2), local infection/skin erosion (2) or painful paresthesia (2). Eight subjects (35%) had their system removed after an average implant time of 30 ± 21 months (range 2 – 61 months), either for inefficacy (4/23), infection (1/23) or both (2/23). One patient requested the removal of the system for psychological reasons despite receiving significant benefit from it.

All 25 patients were reviewed by the headache specialists during the telephone interview. Nineteen patients (76%) were diagnosed with refractory chronic migraine (rCM), 3 (12%) with refractory occipital neuralgia, 1 (4%) with refractory chronic cluster headache and 2 (8%) with other forms of chronic headache (see Table 1). Interestingly, only 7 of the rCM patients were referred with this diagnosis for ONS, while 11/19 were wrongly labelled as occipital neuralgia and 1/19 as chronic headache refractory to medical treatment.

Seventeen patients with a diagnosis of rCM received a permanent ONS system, and all but three had a successful trial before the implant (84% success rate). Two of the subjects with an unsuccessful trial did not proceed to the full implant. One patient, against medical recommendation, decided to undergo full implantation despite limited benefit from the trial and reported a mild benefit (< 50% relief) after 5 years of follow-up.

Nine subjects (53%) reported significant pain relief (> 50% relief in attacks’ intensity and/or frequency) after an average follow-up of 40 ± 27 months (range 11–77 months).

In 5/17 (4 of which with sustained pain relief), migraine attacks originated in the trigeminal nerve distribution, while 11/17 patients had their original pain in the occipital area and of those, 5 reported significant relief over time. It should be noted that in most patients headache pain radiates in both territories as the migraine attacks progress.

Seven of the eight patients who had their system removed were classified as rCM. Five were removed for inefficacy (despite a successful initial percutaneous trial), and one for acquired infection not responding to antibiotic therapy.

Three subjects were classified as refractory occipital neuralgia, with a history of tenderness over the occipital area and temporary pain relief following at least one occipital nerve block with local anaesthetic and/or steroids. All had a successful trial of stimulation and all of them (100%) report significant relief (well over 50% reduction in severity and frequency) after 28, 28 and 31 months of follow/up from the permanent insertion of the ONS system, respectively.

Seven patients (6 rCM and 1 ON) attended a specifically designed, multi-disciplinary, two-week pre-implant programme (PIP). Attending the programme was associated with improved scores across all measured psychological and functional parameters. Statistically significant improvement occurred in the BDI scores, with a mean decrease of 7.4 (95% CI: 2.3 – 12.5), and in the TSK scores, with an average decrease of 8 points (95% CI: 2.2 – 13.8). The analysed population was very small and differences observed between responders and non-responders did not reach statistical significance. However, long-term responders seemed to have higher values of PCS scores before the PIP than non-responders, and were able to decrease their BDI values during the course more than those who failed ONS treatment.

## Discussion

ONS is a promising treatment for some refractory primary headaches, but its role needs further definition. We have presented 6 years ONS experience in two European neuromodulation centres closely working as *twin teams* with tertiary headache centres. Our data is consistent with published studies that suggest ONS has a place in the management of patients with refractory chronic migraine and with refractory occipital neuralgia - but that much work needs to be done to refine patient selection and optimise the treatment. Our analysis has highlighted important specific areas to focus on in the future clinical and research use of ONS.

### The concept of a multidisciplinary approach to refractory headaches

In order to face the clinical challenge of refractory chronic headaches there is a need of at least three different specialists to be involved in the selection of refractory headaches patients as potential candidate for ONS: a referring headache specialist, a pain physician with expertise in neuromodulation and a psychologist with expertise in chronic pain. The presence of a headache specialist with expertise in ICDH-II diagnostic classes must be considered mandatory in future. Many patients included in our analyzed cohort were reclassified when reviewed by a trained headache specialist: only 25% of the patients were correctly labelled as CM at the time of the referral, and only 19% of the subjects originally labelled as occipital neuralgia fulfilled the ICDH-II criteria for this diagnosis. Our results are in line with previous ONS retrospective analysis, where patients reviewed by an headache specialist were often re-coded [26]. As evidenced by the difference between long-term efficacy (53% CM vs 100% ON) and system removal rates (7 patients with CM vs 0 with ON) in our series, correct diagnosis is essential for scientific and economic evaluation of ONS.

Inappropriate use of the words “refractory” and “intractable” might also led healthcare professionals to improperly label patients as “refractory” even if they have not been on an appropriate trial of acute treatments or have never been tried on a preventive medication at an adequate doses for a reasonable period of time [9,15]. Efficacy of onabotulinumtoxinA as a preventive treatment of chronic migraine has been shown in the PREEMPT studies [27] and it should now be added to the list of preventive therapies to be tried before labeling a migraine patient as refractory and offering them invasive treatments. 15 of the patients included in our series had their system implanted before the PREEMPT publication (and therefore did not receive onabotulinumtoxinA treatment), it is possible that some of them might have responded to onabotulinumtoxinA treatment without the need of an ONS implant.

Patients with chronic migraine experience the same complex spectrum of biopsychosocial problems seen in other chronic pain conditions [28,29]. Anxiety, depression, sleep interference, employment interference, relationship interference and decreased physical and social activity are important factors in overall morbidity and should be assessed and addressed. The GSTT subgroup in our data who participated in a pre-implant pain management approach (PIP) experienced improvement across all measured psychosocial domains leading to improved quality of life and health outcomes. However, in our series more patients had a successful implant who did not have a PIP (7/9 vs 3/7), suggesting that while the PIP is efficacious itself, in its current form it may not provide the best preparation of patients for an implant. The current PIP focuses on encouraging patients to manage their pain and maximise activity and does not focus on patient selection for ONS.

Careful assessment of psychosocial domains should lead to improved ONS patient selection and outcomes - this is widely observed recognised in other neuromodulation areas [19,30,31]. Pain duration, psychological distress, pain catastrophising, psychiatric conditions including personality disorders, history of abuse, and significant cognitive deficits are associated with poor outcomes from pain treatments in general [32]. Depression has been identified as the single most important factor predictive of efficacious Spinal Cord Stimulation [33], and other factors including somatization, anxiety, poor coping also predict poor response [34]. Reports on ONS to date have focussed on technical details and patient outcomes have centred on pain scores as a measure of patient benefit [10] and there is an absence of literature looking at patients’ psychosocial and physical status and examining outcomes with quality of life measures.

### Stimulation trial as a reliable predictor for long-term success

A successful temporary trial of stimulation has been considered the best predictor of long-term outcome [35] in different groups of chronic pain patients who are candidates for neuromodulation. However, a positive trial does not guarantee long term success. The two largest, multicenter, prospective trials of spinal cord stimulation for the treatment of chronic pain after spine surgery required a positive trial as key inclusion criteria for patients enrollment [36,37]. Despite an high initial trial to implant ratio (83% in both studies), successful outcome at one year dropped dramatically (55% - 47%) [37,38].

There is no available literature on the ability of a percutaneous trial to predict long-term benefit of ONS implant [39]. Subgroup analysis of data coming from one large RCT of ONS in CM showed that patients who positively responded during a percutaneous trial before the permanent implant reported a decrease in headache days per month significantly greater than those who failed the trial [16]. However, only short term data was published so we do not know if the successful trial predicted long-term benefit. Moreover, we do not know if a longer period of stimulation in those who failed the trial might have resulted in benefit in the longer term. In our series of patients, despite an initial trial success rate of 88%, 7/23 systems were removed due to inefficacy, and only nine subjects (53%) with a diagnosis of chronic migraine reported significant pain relief (>50% relief in attacks’ intensity and/or frequency) after an average follow-up of 40 months. A retrospective review of ONS in heterogeneous headache patient population has been recently published reporting similar data in terms of trial success rate (89%), system efficacy (56%), and long-term benefit in CM patients (42% at an average of 34 months) [40].

Rarely ONS-induced improvements are evident within days, as the neuromodulatory processes involved are believed to occur slowly in different areas of the whole nociceptive system [10]. The reported benefit of a short (7 – 10 days) percutaneous trial might represent a placebo effect in a cohort of subjects who usually have unrealistic expectations on the surgery, after having failed most of the available treatments. The view of the International Headache Society Clinical Trials Subcommittee is that the subjective nature of migraine features and a high placebo effect invalidate open and single- blind trials of any prophylactic intervention and that the number of migraine attacks and number of migraine days should be collected prospectively for an interval of time long enough to be compared with a prospective baseline of at least 1 month [41]. A one or two weeks percutaneous ONS trial will not satisfy this standard. Furthermore, when a one-month, semi-permanent, tunnelled trial was employed to test ONS system efficacy before implantation, the long-term outcome in CM patients was still only 47%, despite an accurate evaluation of trial outcomes through specific pain questionnaires [26].

Therefore, the use of a trial test of ONS is now highly questionable. Its ability to select long-term responders appears poor and with >80% of patients going on to full implantation anyway, a trial poses additional risk and inconvenience for patients and an economic burden to the health care system.

### Long-term treatment efficacy

Neuromodulation is an invasive and expensive treatment, and should be reserved for specific subset of chronic pain patients following evidence-based guidelines [42]. 350 patients have been enrolled in three large, industry sponsored, randomized control trials in the efforts to evaluate safety and efficacy of ONS to treat rCM [12,13,16]. Two found no significant support for an adequate therapeutic effect (responders defined as 50% reduction in headache days per month), and the other found only a moderate benefit (responders defined as 30% improvement in pain) in 39% of the treated subjects. Different study designs, with controversial end-point choices, do not allow a direct comparison of the trials’ results. Furthermore, no conclusions on long-term treatment efficacy can be drawn as only 3 months follow/up data have been reported to date. In our patients the average time of system removals for inefficacy is around 23 months (range 2 – 54).

An analysis of the available ONS literature reported long-term implant response rate is high (88% to 100%) when peripheral stimulation is performed to elicit paresthesia in the whole painful area, compared to a low response rate (40%) in those studies reporting non-concordant paresthesia [43]. Some authors hypothesized that the combined stimulation of areas innervated by both the occipital nerves (ON) and supraorbital nerves (SON) might benefit those patients who perceived pain in a hemicephalic or global extent [43,44]. Interestingly, in our series we found no differences among the patients who reported Excellent/Good paresthesia in terms of long-term positive outcome (64% vs 66%), and 4 out of 5 patients with migraine origin in the trigeminal area had good long-term outcome. Moreover two patients had a supraorbital lead added later on in the attempt of increase paresthesia coverage and system efficacy, but only one of them reported significant benefit. As adding supraorbital leads increases surgical times and complexity, a carefully designed trial is warranted to establish the long-term benefit of this new approach.

### Hardware-related complications

Currently available ONS technology, originally designed for epidural use, is associated with troublesome complications when used subcutaneously for ONS. Skin erosion, lead breakage, lead migration, and pain around the battery site can occur. These are not only direct adverse events for the patient, but also impact on ONS efficacy, and dramatically increase health care expenditure as further surgical procedures and new equipment are often required. In our series, almost 43% of the patients required at least one surgical revision to treat such problems. In 90% of cases leads or the intermediate connections were the culprit. Similar numbers have been reported in another recent retrospective review of ONS in heterogeneous headache patient population, with 58% of patients needing a surgical revision [40]. In the larger RCTs, where only 3 months data have been disclosed, surgical revision rates were already between 19% [13] and 37% [12].

Lead migration and lead breakage, major causes of ONS-related surgical revision, are related to repeated lead and extension traction events due to the high mobility of the implanted area. Over the years, some authors have described techniques to minimize these complications. Bennett suggested securing each lead ipsilaterally to the lateral pocket fascia using 2 suture sleeves separated by a strain relief loop, and anchoring each sleeve to the fascia with 3 sutures and intraluminal medical adhesive [16]. Franzini et al. recommend securing the distal end of the lead to the lateral portion of the superficial cervical fascia (with two additional skin incisions) to prevent lead migration and report no displacement at 1 year follow-up in 17 patients [45]. Additional strain relief loops are recommended at the upper thoracic level (T2- T4), at the implantable pulse generator (IPG), and at any other incisions [16]. Finally, IPG implantation sites other than the traditional gluteal region may have the advantage of less pathway length change during patient movement. Thus, infraclavicular and low abdomen IPG sites may result in less lead migration/rupture [46]. This literature reveals that specialist expertise by the neuromodulator is important factor in outcome.

### Limitations

Our audit has several weaknesses. Its design is flawed by the well-known limitations of retrospective case-series studies [47]. Lead/anchor technology and our surgical technique have evolved so some of the problems we have highlighted are already being addressed. Different measures were collected over the years, and our choice of using patients’ subjective report of headache’s intensity/frequency reduction to define long-term success is not highly robust. Any prospective trial should now endorse the outcome measures defined by Task Force of the International Headache Society Clinical Trials Subcommittee [41]. Finally, we couldn’t collect enough information to report and comment on medication-overuse headache.

## Conclusions

Our audited series of 25 patients treated with ONS in two tertiary neuromodulation centers is consistent with literature suggesting that ONS is a therapeutic option for patients with refractory chronic migraine (9 of 17 patients reporting >50% reduction in headache frequency and or intensity at long-term follow up), and refractory occipital neuralgia (all patients reporting >50% reduction in pain frequency and or intensity at long-term follow up).

There is a need to refine patient selection for ONS and ensure optimal medical, psychological and surgical management at all stages - a multidisciplinary team comprising of headache, psychology, and neuromodulation specialists is essential for this. Such teams should be used in future randomized controlled trials with long-term follow-up to further determine the place for ONS in refractory chronic headache management and improve patient outcomes.

## Competing interests

SP has received travel reimbursement from Medtronic and Nevro Corp.

AA has received travel sponsorship and speaker fees from Medtronic and Nevro Corp, and he is the principal investigator in separate studies sponsored by Medtronic and Nevro Corp.

RA has received travel reimbursement from Medtronic and Nevro Corp.

TS has received travel sponsorship and speaker fees from Medtronic and Nevro Corp.

PM has received travel grants from Nevro Corp and St Jude Medical.

AN, EC, VB and GL do not declare any competing interest.

## Authors’ contributions

SP designed the study, supervised the data collection, performed data analysis and drafted the initial manuscript. AA, RA, TS and SP performed the surgical procedures. AN, GL and PM reviewed patient’s notes and diagnosis. VB and EC collect data and took part in data analysis. All authors revised the manuscript. All authors read and approved the final manuscript.
